# Plating human iPSC lines on micropatterned substrates reveals role for *ITGB1* nsSNV in endoderm formation

**DOI:** 10.1016/j.stemcr.2021.09.017

**Published:** 2021-10-21

**Authors:** Alice Vickers, Mukul Tewary, Anna Laddach, Martina Poletti, Vasiliki Salameti, Franca Fraternali, Davide Danovi, Fiona M. Watt

**Affiliations:** 1 Centre for Stem Cells and Regenerative Medicine, King's College London, Guy's Hospital, Floor 28, Tower Wing, Great Maze Pond, London SE1 9RT, UK; 2 Randall Centre for Cell and Molecular Biophysics, King's College London, New Hunt's House, Great Maze Pond, London SE1 9RT, UK; 3 Development and Homeostasis of the Nervous System Laboratory, The Francis Crick Institute, London NW1 1AT, UK; 4 Earlham Institute, Norwich Research Park, Norwich NR4 7UZ, UK; 5 Quadram Institute, Norwich Research Park, Norwich NR4 7UZ, UK; 6 bit.bio, Babraham Research Campus, The Dorothy Hodgkin Building, Cambridge CB22 3FH, UK

**Keywords:** cell adhesion, genomics, high-content imaging, differentiation

## Abstract

Quantitative analysis of human induced pluripotent stem cell (iPSC) lines from healthy donors is a powerful tool for uncovering the relationship between genetic variants and cellular behavior. We previously identified rare, deleterious non-synonymous single nucleotide variants (nsSNVs) in cell adhesion genes that are associated with outlier iPSC phenotypes in the pluripotent state. Here, we generated micropatterned colonies of iPSCs to test whether nsSNVs influence patterning of radially ordered germ layers. Using a custom-built image analysis pipeline, we quantified the differentiation phenotypes of 13 iPSC lines that harbor nsSNVs in genes related to cell adhesion or germ layer development. All iPSC lines differentiated into the three germ layers; however, there was donor-specific variation in germ layer patterning. We identified one line that presented an outlier phenotype of expanded endodermal differentiation, which was associated with a nsSNV in *ITGB1.* Our study establishes a platform for investigating the impact of nsSNVs on differentiation.

## Highlights

• Healthy donor iPSCs differentiate into radial germ layers on micropatterns
• Differentiation can be quantified using high-content image analysis
• iPSC lines show donor-specific variation in germ layer patterning
• A deleterious nsSNV in *ITGB1* is associated with expanded endoderm formation

## Graphical abstract

**Figure:**
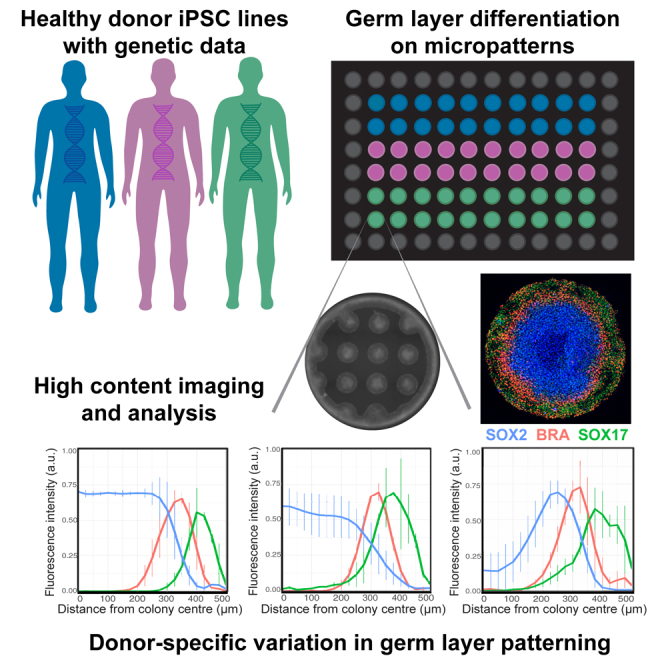

## Teaser

In this study, Vickers and colleagues combine micropatterning and high-content image analysis of healthy donor iPSC lines to identify genetic variants that influence cellular differentiation. iPSC lines displayed donor-specific variation in germ layer patterning. Outlier cell lines were identified, including one line that displayed expanded endoderm differentiation and was associated with a deleterious nsSNV in *ITGB1*.

## Introduction

Human induced pluripotent stem cells (iPSCs) provide an accessible resource for the *in vitro* study of human development and disease mechanisms and have demonstrated their potential to provide patient-specific cells for regenerative medicine (Fatehullah et al., 2016; Liu et al., 2018; Mandai et al., 2017; Park et al., 2008; Takahashi et al., 2007; Yamanaka, 2020). However, substantial phenotypic variation has been observed between iPSC lines, with different iPSC lines showing a bias or even deficiency in differentiating toward certain lineages (Chichagova et al., 2020; Hu et al., 2010; Koyanagi-Aoi et al., 2013; Ortmann and Vallier, 2017). Studies based on multiple iPSC lines from the same donor, different reprogramming methods, and isogenic iPSC lines from different source cell types have often found that the genetic background of the donor is a major contributor to iPSC variability (Bock et al., 2011; Burrows et al., 2016; Rouhani et al., 2014).

The Human Induced Pluripotent Stem Cell Initiative (HipSci) was established to create a large, high-quality reference panel of iPSCs with accompanying genetic, proteomic, and phenotypic data. Genetically diverse, large-scale collections of iPSCs such as HipSci have enabled the identification of genetic factors that influence gene expression and cellular phenotypes in both pluripotent and differentiated cells (Bonder et al., 2021; Carcamo-Orive et al., 2017; Kilpinen et al., 2017; Panopoulos et al., 2017; Schwartzentruber et al., 2018; Warren et al., 2017). Using the HipSci resource, we have previously combined cell-based assays, high-content imaging, and genome sequencing datasets to identify rare, deleterious, non-synonymous single nucleotide variants (nsSNVs) in genes related to cell adhesion that are associated with outlier iPSC phenotypes in the pluripotent state (Vigilante et al., 2019).

In this study, we aimed to generate an *in vitro* model that reflects *in vivo* genetics to map normal genomic variation to more complex cell behaviors. For this, we employed a recently described micropatterning-based differentiation platform (Tewary et al., 2019). Building upon our previous study (Vigilante et al., 2019), we selected iPSC lines from the HipSci cell bank that harbor rare and deleterious nsSNVs in genes related to cell adhesion and/or germ layer differentiation. We developed a novel, custom-built image analysis pipeline that quantifies differentiation phenotypes with spatial resolution. Our study establishes an *in vitro* method to quantify iPSC differentiation propensity and investigate the genetic contribution to inter-individual phenotypic variability.

## Results

### Development of an *in vitro* micropatterned platform for germ layer differentiation of iPSCs

**Figure 1:**
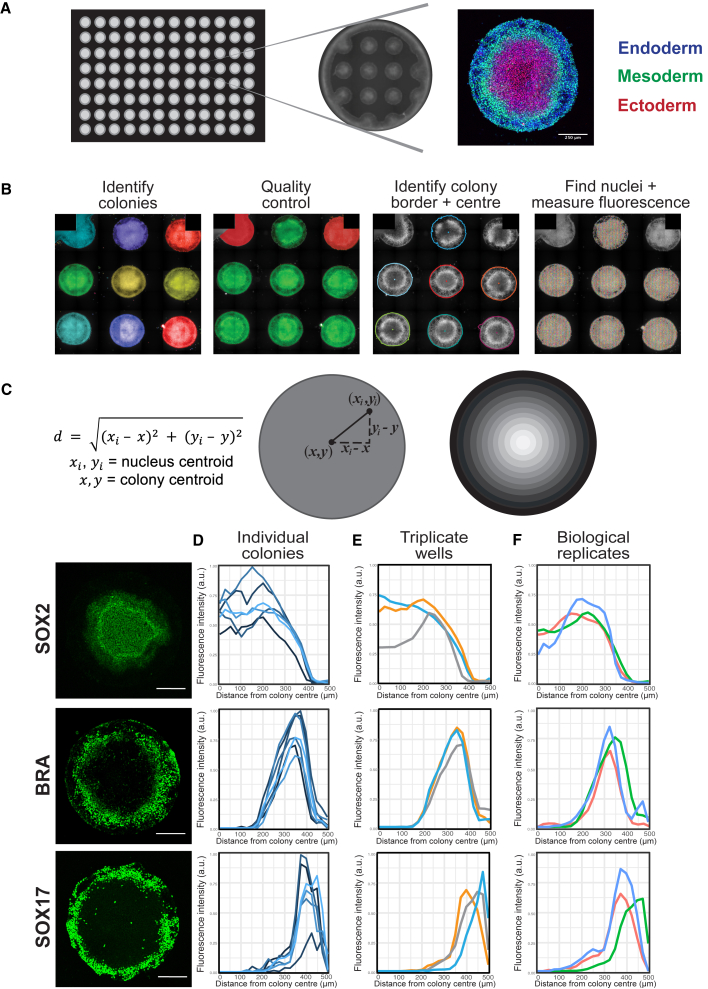
High-throughput quantification of iPSC germ layer differentiation on micropatterned substrates (A) Representative images of (left to right): one 96-well plate; one well containing micropatterned substrates surrounded by an inert substance that cells cannot adhere to; one micropatterned substrate containing cells labeled with antibodies to markers of the three germ layers (SOX2, endoderm; BRA, mesoderm; SOX17, ectoderm) with DAPI counterstain (white). (B) The Harmony script identifies colonies within each well (colors represent individual colonies), then performs quality control based on morphology. Incomplete colonies at the edge of the well are discarded (red), while complete colonies are qualified for quantification (green). For qualifying colonies, the outer border and geometrical center are defined, the nuclei are segmented, and the fluorescence intensity of the proteins of interest localized within each nucleus are measured. (C) The Harmony data are imported into R where a script is written to calculate the distance of each nucleus from the colony centroid (D) using the nucleus centroid (*x*_*i*_, *y*_*i*_) and colony centroid (*x*, *y*), which was used to assign nuclei into radial bins (B). (D–F) Antibody labeling to detect protein expression of the germ layer markers SOX2 (ectoderm), BRA (mesoderm), and SOX17 (endoderm). Left-hand panels show representative colonies. Within each nucleus, the fluorescence intensity of the protein marker was normalized to DAPI intensity. These values were used to calculate the average protein expression within each bin for each colony, which was then normalized to the maximum expression value within the well. Protein expression was plotted as a function of distance from the colony center (μm). Plots were generated for (D) individual colonies in one well, where each line represents protein expression within a colony; (E) triplicate wells, where each line represents average protein expression across colonies from one technical replicate (i.e., one well); and (F) biological replicates, where each line represents average protein expression across technical replicates from one experiment. Scale bars, 250 μm.

We employed a previously described bioengineered 96-well plate micropatterning platform that geometrically confines PSCs on 1000-μm diameter circular micropatterned islands, which generate reproducible spatially ordered germ layer fates in response to BMP4 and NODAL (Figures 1A–1C) (Tewary et al., 2019). SOX2 expression was used as a marker of ectoderm, BRACHYURY (BRA) as a mesoderm marker, and SOX17 as a marker of endoderm (Tewary et al., 2019). As reported previously (Tewary et al., 2017; Warmflash et al., 2014), SOX17 and BRA were expressed by cells in the outer regions of the colonies, whereas SOX2-expressing cells were in the center (Figures 1D–1F). In contrast, undifferentiated colonies maintained in basal media conditions without BMP4 and NODAL co-expressed SOX2 and OCT4, which indicated that these cells remained pluripotent (Figure S1A).

The format of the platform enabled the use of automated high-content image analysis methods. We custom-built an analysis pipeline to (1) identify each micropatterned colony, (2) select colonies quality controlled on colony area and roundness, (3) determine the outer border and geometrical center of each colony, and (4) segment individual nuclei based on the expression intensity in the DAPI channel and measure the fluorescence intensity of the proteins of interest localized within each nucleus (Figure 1B). Colonies that were not round because they did not fill the entire micro-pattern and patterns that were not round because they were printed at the edge of a well were automatically excluded. The total number of readable colonies per well was four to seven.

The image analysis pipeline calculated the distance of each nucleus from the colony centroid using the equation in Figure 1C. Each colony was divided into 20 concentric rings spaced 25 μm apart, with nuclei assigned to the rings based on their position relative to the colony center (Figure 1C). Thus, the pipeline automates the analysis of iPSC germ layer phenotypes, including quantification of total protein expression per colony and spatial patterning.

To evaluate whether the pipeline could provide quantitative comparisons of the differentiation capacities of different iPSC lines, we first investigated the level of variation in differentiation of a single iPSC line, uoxz_4, from a healthy donor. This line lacks any nsSNVs in genes related to cell adhesion or germ line differentiation. The mean protein expression of each germ layer marker on each micropatterned substrate was normalized to the highest value per well and plotted as a function of distance from the colony center (Figure 1D). The background fluorescence of cells that did not express the protein being analyzed was subtracted as shown in Figure S1B. Data from triplicate wells within an experiment provided technical replicates (Figure 1E), while data from three independent experiments provided biological replicates (Figure 1F). This provided up to 100 colonies per protein marker for analysis. The spatial patterning of each germ layer was compared between technical and biological replicates of the iPSC line uoxz_4 using a Kolmogorov-Smirnov test. This showed that the spatial patterning of each germ layer was reproducible between technical and biological replicates (p > 0.05) (Figures 1D–1F and S2A; Table S1).

### Differentiation of control iPSC lines

**Figure 2:**
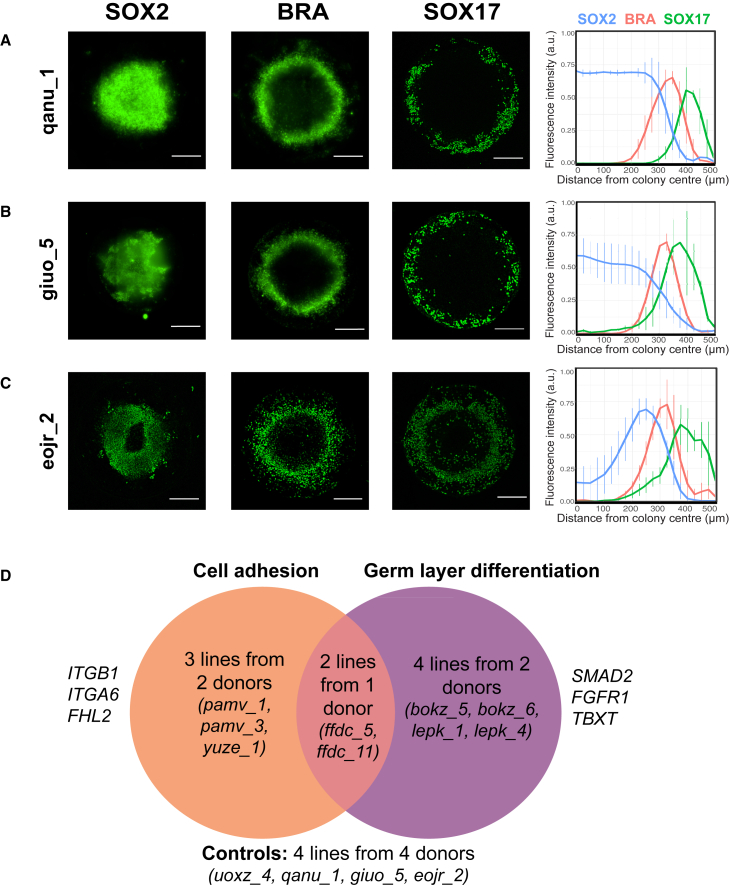
iPSCs differentiated on micropatterns form spatially ordered germ layers (A–C) The iPSC lines qanu_1, giuo_5, and eojr_2 were seeded at a density of 60,000 cells/well on micropatterned substrates overnight. Cells were treated with 50 ng/mL BMP4 and 100 ng/mL NODAL for 48 h before fixation and were stained with antibodies to detect SOX2 (ectoderm), BRA (mesoderm), and SOX17 (endoderm). Plots show average radial trends of SOX2, BRA, and SOX17 expression for each cell line. Error bars represent SD. Representative confocal images are shown from n = 3 experiments, each performed in triplicate (scale bars, 250 μm). (D) Genome sequencing data for over 700 iPSC lines available through HipSci were analyzed to identify cell lines with rare and deleterious nsSNVs in genes related to cell adhesion (e.g., *ITGB1*, *ITGA6*, and *FHL2*) and/or germ layer differentiation (e.g., *SMAD2*, *FGFR1*, and *TBXT*) and control iPSC lines with no known nsSNVs in cell adhesion or germ layer differentiation genes. Clonal iPSC lines from the same donor are denoted by the same four-letter word with a unique number.

Using our differentiation and analysis platform, we compared a panel of iPSC lines from three additional healthy donors, lacking any nsSNVs in genes related to cell adhesion or germ line differentiation. This enabled us to characterize the reproducibility of germ layer differentiation phenotypes within and between cell lines from different donors. Following differentiation with 50 ng/mL BMP4 and 100 ng/mL NODAL for 48 h, we evaluated spatial patterning of SOX2, BRA, and SOX17 (Figures 2A–2C).

We observed variation in the quantity and spatial patterning of the germ layers between iPSC lines from different donors. In qanu_1, the peak of SOX2 expression was detected up to 300 μm from the colony center (Figure 2A). In contrast, SOX2 expression was highest <250 μm from the colony center in giuo_5 (Figure 2B) and was highest in a ring 100–300 μm from the colony center in eojr_2 (Figure 2C). BRA expression was detected >200 μm from the colony center in qanu_1, giuo_5, and eojr_2, either as a distinct ring, as in giuo_5 (Figure 2A), or diffuse patterns, as in qanu_1 and eojr_2 (Figures 2B and 2C). SOX17 expression was detected in a ring at the colony periphery in qanu_1, giuo_5, and eojr_2, which was distinct from the BRA+ region in qanu_1 (Figure 2A), but partially overlapped with BRA expression in giuo_5 and eojr_2 (Figures 2B and 2C). The majority of colonies displayed discontinuities in the ring of SOX17 expression, which is consistent with previous studies (Tewary et al., 2017; Warmflash et al., 2014). As in the case of uoxz_4 (Figures 1D–1F), we observed reproducible phenotypes between technical and biological replicates in each individual cell line (p > 0.05) (Figures 2A–2C and S2; Table S1).

### Cell line selection

We previously identified 103 rare, destabilizing, and deleterious nsSNVs in a subset of healthy donor cell lines from the HipSci resource (Vigilante et al., 2019). These genes encode proteins associated with cell adhesion, including integrins, cytoskeleton components, and extracellular matrix (ECM) proteins (Vigilante et al., 2019). The nsSNVs were present in 19 out of the 29 cell lines that displayed outlier phenotypes in the pluripotent state when seeded for 24 h on different fibronectin concentrations (Vigilante et al., 2019). Cell adhesion is a key determinant of cell behaviors such as migration, cell-cell contact, and communication, as well as somatic stem cell differentiation (Adams and Watt, 1993; Ramos et al., 1996). We therefore hypothesized that the identified nsSNVs could influence more complex *in vitro* cellular behaviors, specifically the differentiation of germ layer fates.

For our analysis, we searched for further genes with nsSNVs. Exome sequencing datasets for over 700 lines available through HipSci identified rare and deleterious nsSNVs in 124 genes related to germ layer differentiation. nsSNVs were classified as rare if present in fewer than five of the HipSci cell lines. nsSNVs were classified as deleterious to protein function based on the computational model Condel. This identified 270 nsSNVs that were present in 229 lines from 176 donors, details of which can be found in Table S2. The list includes the 103 nsSNVs identified previously (Vigilante et al., 2019).

Based on our genetic analysis, we selected 13 iPSC lines from nine healthy donors for phenotypic characterization. The lines fell into four categories (Figure 2D). We selected three lines from two donors that were phenotypic outliers when plated on fibronectin and had nsSNVs in cell adhesion-associated genes (Vigilante et al., 2019). We chose four lines from two donors with deleterious nsSNVs in genes related to germ layer differentiation. These genes encode nodes along key signal transduction pathways involved in germ layer specification, such as FGFR1, SMAD2, and BRA (encoded by *TBXT*). The third category comprised two lines from one donor that were phenotypic outliers on fibronectin and had deleterious nsSNVs in both germ layer specification- and cell adhesion-associated genes (Vigilante et al., 2019). The identified nsSNVs were mapped to the corresponding protein domains (Figure S3). Finally, we included the four control iPSC lines, each from a different donor, characterized in Figures 1 and 2.

### Genetic contribution to variation in germ layer differentiation of iPSC lines

**Figure 3:**
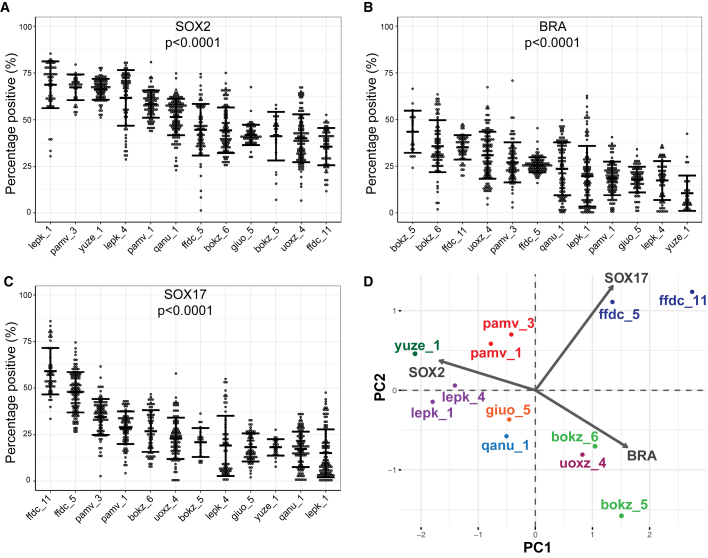
Analysis of germ layer protein expression in iPSC lines (A–C) Quantification of (A) SOX2, (B) BRA, and (C) SOX17 protein expression in all iPSC lines tested plotted as the percentage of the total number of cells within the colony that expressed the protein of interest. Each data point represents an individual colony (number of colonies analyzed provided in Table S3). Colonies were pooled from three independent experiments. Error bars represent mean ± SD. p values represent the difference between all iPSC lines tested for each germ layer marker and were calculated using Kruskal-Wallis test with Dunn's multiple comparison post hoc test. (D) Principal component analysis of germ layer protein expression in all iPSC lines tested.

We quantified protein expression of each germ layer marker within individual colonies for all cell lines tested using the image analysis pipeline. The results are presented as the percentage of the total number of cells within each colony that expressed the protein of interest, which controls for variation in the number of cells per colony (Tewary et al., 2019). There was considerable variation in expression of germ layer proteins between cell lines from different donors (Figures 3A–3C). To identify cell lines that were phenotypic outliers, mean protein expression in each individual cell line was compared with the mean expression in all other cell lines pooled together, with p < 0.001 considered significant. The iPSC lines lepk_1, yuze_1, and ffdc_11 were identified as outliers for SOX2 expression, yuze_1 was an outlier for BRA expression, and ffdc_5 and ffdc_11 were outliers for SOX17 expression (Figures 3A–3C).

The use of independently derived clonal lines from the same donor was used to confirm the genetic contribution to cell phenotypes (Vigilante et al., 2019). We performed a principal component analysis using protein expression data (percentage of positive cells) for the three differentiation markers. Each clonal line from a single donor fell within the same region, which indicates similar expression behavior of SOX2, BRA, and SOX17 (Figure 3D). This suggests that there is indeed a genetic contribution to germ layer differentiation.

We also found that SOX2 expression was moderately negatively correlated with BRA expression (*r* = −0.59) and SOX17 expression (*r* = −0.48) (Figures S4A and S4B). Thus, for example, lepk_1 had the highest %SOX2-positive cells and the lowest %SOX17-positive cells (Figures 3A and 3C). In contrast, there was a moderate positive correlation between BRA and SOX17 expression (*r* = 0.50) (Figure S4C). In most experiments, a control iPSC line, eojr_2, was included to account for technical variability between experiments. The variation seen in the control cell line replicates was less than the inter-donor variation (Figures S4D–S4F).

### Differentiation of iPSC lines with germ layer differentiation- and/or cell adhesion-related nsSNVs

We next investigated spatial patterning of the germ layer markers within the selected cell lines. Three of the donors had deleterious nsSNVs in cell adhesion genes (Figure 2D). The cell line yuze_1 is a phenotypic outlier in the pluripotent state, since yuze_1 cells display reduced cell attachment and spreading when plated on fibronectin (Vigilante et al., 2019). Yuze_1 harbors a rare and deleterious nsSNV in *ITGA6* (Vigilante et al., 2019). Similarly, the cell line ffdc_11 displays an outlier phenotype of reduced cell attachment and spreading (Vigilante et al., 2019). Ffdc_11 and a different clonal line from the same donor, ffdc_5, harbor rare and deleterious nsSNVs in *ITGB1* and *TBXT*. We also identified a rare, deleterious nsSNV in the gene *FHL2* in the clonal iPSC lines pamv_1 and pamv_3. FHL2 interacts with cell membrane proteins such as integrins and focal adhesion kinase (FAK) (Gabriel et al., 2004; Samson et al., 2004).

**Figure 4:**
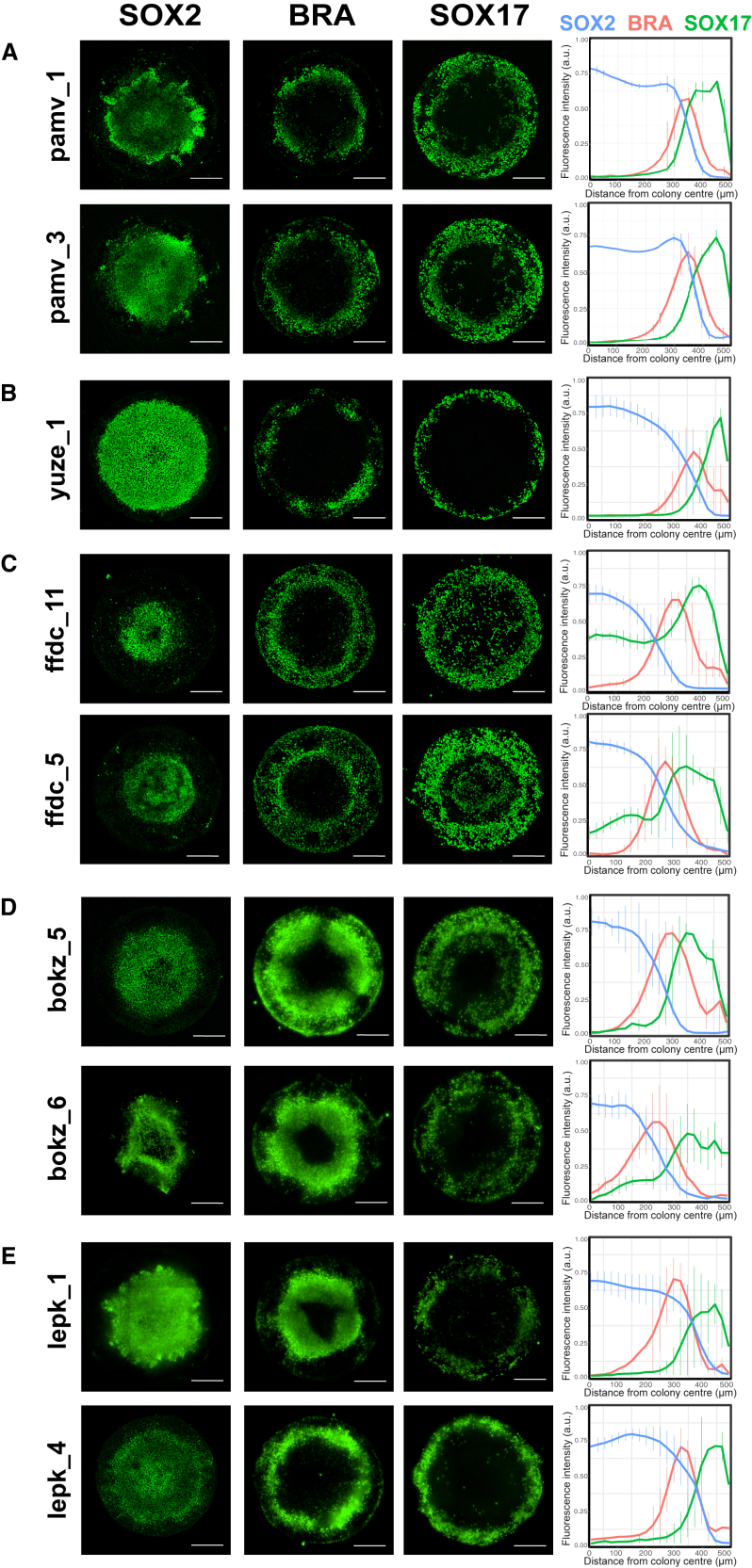
Differentiation of selected iPSC lines with germ layer differentiation- and/or cell adhesion-related nsSNVs (A–E) Representative immunofluorescence images and radial trend plots of SOX2, BRA, and SOX17 expression for the iPSC lines tested. Data are shown from n = 3 experiments, each performed in triplicate. Error bars represent mean ± SD. Scale bars, 250 μm.

We tested cell lines from donors pamv, yuze, and ffdc to understand whether the identified polymorphisms could influence their differentiation propensity. The lines pamv_1 and pamv_3 displayed high overall SOX17 expression (28.6% and 34.5%, respectively) (Figure 3C), with the peak of SOX17-positive cells distributed 300–500 μm from the colony center (Figure 4A). SOX2 expression was highest 0–300 μm from the colony center (Figure 4A). The SOX2 spatial profiles for pamv_1 and pamv_3 were identified as outliers compared with the control line uoxz_4 (see experimental procedures for classification of outliers). Line yuze_1 was a phenotypic outlier for SOX2 spatial patterning, which was expressed 0–400 μm from the colony center (Figure 4B). Lines ffdc_5 and ffdc_11 were outliers for SOX17 spatial patterning, which extended into the colony center where cells co-expressed FOXA2, but were distinct from the SOX2+ population (Figures 4C, S5A, and S5B).

We next tested the selected iPSC lines with rare, deleterious nsSNVs in genes related to germ layer differentiation (Figure 2D). These included bokz_5 and bokz_6, which harbor a deleterious nsSNV in *FGFR1*, and presented high expression of BRA (43.5% and 35.7%, respectively) (Figures 3B and 4D). Lines lepk_1 and lepk_4 harbor a deleterious nsSNV in *SMAD2*, an effector of NODAL signaling required for mesendodermal specification. Both lepk_1 and lepk_4 were outliers for SOX2 spatial patterning, which was highest 0–350 μm from the colony center (Figure 4E).

### Investigation of extrinsic and intrinsic drivers of differentiation

**Figure 5:**
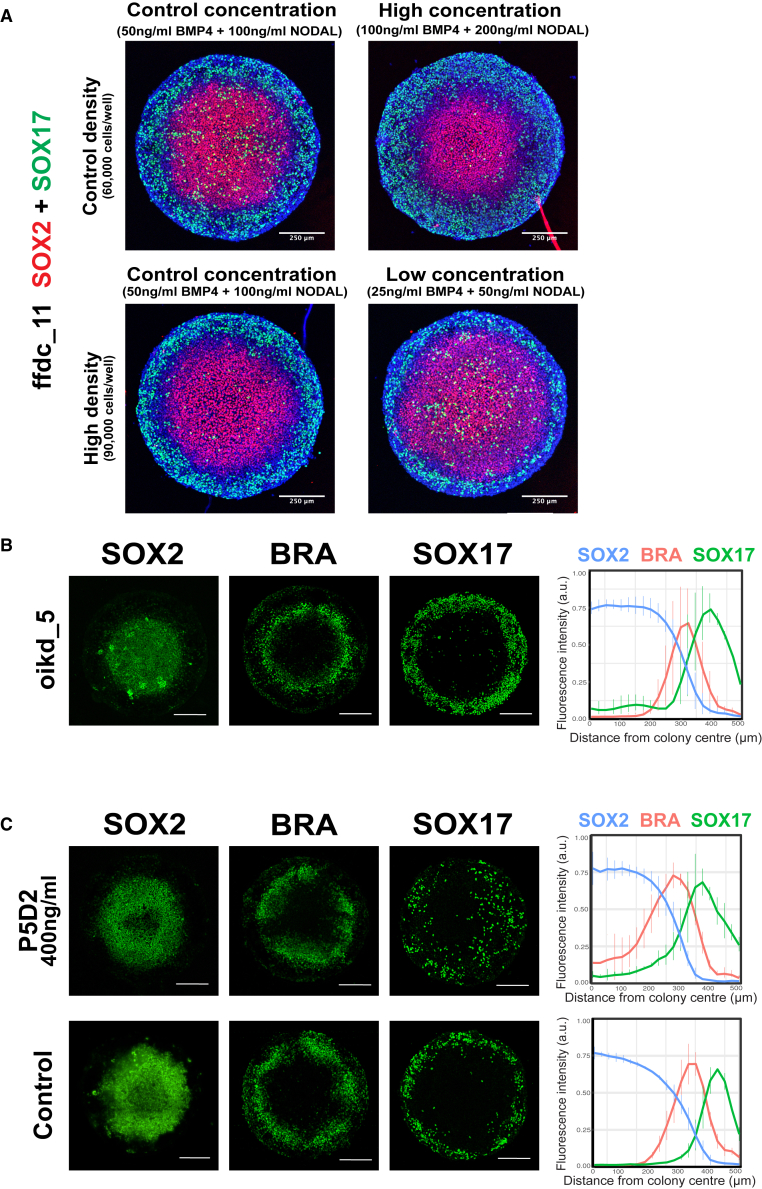
Investigation of extrinsic and intrinsic drivers of differentiation (A) The iPSC line ffdc_11 was seeded at a control (60,000 cells/well) density or high (90,000 cells/well) density and differentiated with control, low, or high concentrations of BMP4 and NODAL. Cells were fixed and stained with antibodies to detect SOX2 (red) or SOX17 (green), plus DAPI (blue). (B) Representative immunofluorescence images and radial trend plots of SOX2, BRA, and SOX17 expression for the iPSC line oikd_5, which harbors a rare, deleterious nsSNV in *TBXT*. (C) Representative immunofluorescence images and radial trend plots of SOX2, BRA, and SOX17 expression for the iPSC line uoxz_4 cultured in the presence of an inhibitory anti-ITGB1 antibody (P5D2, 400 ng/mL) or control conditions. Scale bars, 250μm. p values were calculated using the Kolmogorov-Smirnov test.

We next sought to investigate extrinsic (i.e., cell density and exogenous growth factor concentration) or intrinsic (i.e., genetic variants) drivers of the outlier phenotype observed in the ffdc iPSC lines. Previous studies have shown that mesendodermal fates are restricted to the colony edge due to expression of the secreted BMP inhibitor Noggin (NOG) in the colony center (Etoc et al., 2016; Tewary et al., 2017; Warmflash et al., 2014). However, endoderm expression expanded into the colony center in ffdc_5 and ffdc_11. We therefore hypothesized that the outlier phenotype could be due to a lack of endodermal inhibitors in the colony center and could be rescued by increasing the expression of such inhibitors, either through increasing cell density or exogenous growth factor concentrations. As predicted, the outlier phenotype was rescued by increasing the concentrations of BMP4 and NODAL or by increasing cell density (Figure 5A). In contrast, the outlier phenotype persisted when the cells were plated at the higher cell density and cultured with lower concentrations of BMP4 and NODAL (Figure 5A). Together, these results suggest that endogenous BMP4-NOG signaling may be impaired in the ffdc iPSC lines.

Lines ffdc_5 and ffdc_11 harbor deleterious nsSNVs in the genes *TBXT*, which encodes BRA, and *ITGB1*. To investigate whether these polymorphisms contribute to the outlier SOX17 phenotype, we first searched for further iPSC lines that harbored nsSNVs in *TBXT* or *ITGB1*. We identified oikd_5, which harbors a deleterious nsSNV in *TBXT* and had not been included in the initial panel of HipSci cell lines for phenotypic characterization (Figure 5B). When differentiated on micropatterns, oikd_5 did not display the expanded SOX17 expression observed in ffdc_5 and ffdc_11 (Figure 5B). Therefore an nsSNV in *TBXT* is not sufficient to account for the outlier phenotype of ffdc_5 and ffdc_11.

Besides ffdc_5 and ffdc_11, no other iPSC lines in the HipSci bank harbored a deleterious SNV in *ITGB1*. As an alternative strategy to test the effect on differentiation, we cultured the control line uoxz_4, which does not have deleterious nsSNVs in cell adhesion or gastrulation-related genes, on micropatterned substrates in the presence of an adhesion inhibitory anti-ITGB1 antibody (P5D2) (Byron et al., 2009). P5D2 treatment has previously been shown to phenocopy the effect of a dominant negative integrin mutation in human epidermal stem cells (Haase et al., 2001; Zhu et al., 1999). We found that 100 ng/mL P5D2 had no effect on germ layer phenotype, while 500 ng/mL P5D2 caused the colonies to collapse (Figure S5C). When cultured with 400 ng/mL P5D2, uoxz_4 exhibited an expansion in endoderm expression toward the colony center (Figure 5C). The distribution of SOX17 expression differed significantly between the conditions (p = 0.002, Kolmogorov-Smirnov test), whereas there was no significant difference in the distribution of SOX2 (p = 0.4) or BRA (p = 0.09). This suggests a contribution of the nsSNV in *ITGB1*, and therefore cell adhesion, to the outlier endodermal phenotype.

## Discussion

iPSC lines have been shown to display variable propensity to differentiate, including preference toward certain lineages, loss of differentiation capacity, and teratoma formation (Keller et al., 2018). Recent analyses of large panels of iPSCs from a diverse range of donors, including multiple clonal lines from the same donor, have established that genetic diversity between donors drives variability in cellular phenotypes (Carcamo-Orive et al., 2017; Kilpinen et al., 2017; Panopoulos et al., 2017). In this study, we sought to identify specific genetic variants that influence iPSC differentiation.

We found that micropatterned cell colonies were amenable to automated high-content imaging, allowing quantification of differentiation phenotypes, including the spatial patterning of germ layer proteins. Our approach has several advantages over teratomas (e.g., TeratoScore [Avior et al., 2015], embryoid bodies [e.g., PluriTest; Müller et al., 2011], and ScoreCard [Tsankov et al., 2015]) in terms of speed and reproducibility. Three-dimensional (3D) embryo-like structures generated from ESCs more closely recapitulate aspects of human *in vivo* development than two-dimensional (2D) micropatterns (Harrison et al., 2017; Moris et al., 2020; Sozen et al., 2018). However, 3D models can display poor reproducibility and can be difficult to image using high-content microscopy owing to their size and opacity (Alsehli et al., 2020). In terms of scalability, the rate-limiting step of our platform is maintaining multiple cell lines in culture prior to plating on the micropatterns, which could be mitigated if cells could be seeded directly on thawing.

We found that all iPSC lines tested could differentiate into the three embryonic germ layers in response to BMP4 and NODAL. However, iPSC lines from different donors showed variable differentiation phenotypes. This is consistent with previous studies showing that inter-individual genetic variation between iPSC lines accounts for variable differentiation propensity in multiple cell lineages (Boulting et al., 2011; Kajiwara et al., 2012; Kyttälä et al., 2016; Mills et al., 2013; Nasu et al., 2013).

The iPSC lines tested were derived from healthy adult donors, and therefore the perturbation in germ layer patterning we observed was clearly not linked to developmental abnormalities. However, the extent to which human embryonic development differs between individuals is largely unknown. *In vitro* studies may help reveal cellular phenotypes that are difficult to identify *in vivo* (Cuomo et al., 2020) and are informative for the *in vitro* differentiation of iPSCs for research and clinical applications.

We used our platform to investigate potential intrinsic and extrinsic drivers of cellular phenotypes. Previous studies using micropatterned substrates to generate organized PSC-derived germ layer fates have shown that BMP inhibitors expressed in the center of the colony restrict mesendodermal expression to the colony edge (Etoc et al., 2016; Tewary et al., 2017; Warmflash et al., 2014). This includes the BMP inhibitor NOG, whose expression is upregulated in response to BMP4 in a reaction-diffusion network (Etoc et al., 2016; Tewary et al., 2017). We found that increasing either the cell density or concentrations of BMP4 and NODAL rescued the outlier phenotype observed in the cell line ffdc and restricted endodermal expression to the colony edge. This might be due to an increase in expression of BMP inhibitors such as NOG in the colony center under these conditions. The variability in SOX17 expression profiles between cell lines from different donors may reflect inter-individual variation in the BMP-NOG signaling network, which may be more sensitive to environmental perturbations than other germ layer fates.

Cell fate acquisition is regulated by cell-ECM interactions, cell-cell communication, and internal molecular signaling mechanisms (Adams and Watt, 1993; Arnold and Robertson, 2009; Ramos et al., 1996). We previously identified an association between nsSNVs in cell adhesion genes and outlier cell behaviors in the pluripotent state (Vigilante et al., 2019). Line ffdc, which was a phenotypic outlier in the pluripotent state, was also an outlier in the current study. Consistent with our previous findings (Vigilante et al., 2019), we mapped the outlier phenotype to a deleterious nsSNV in *ITGB1*. Although the outlier endodermal phenotype of ffdc could be detected by visual inspection of SOX17 immunofluorescent labeling, variation in germ layer protein expression in other iPSC lines was not obvious and required quantification. Our analysis pipeline can be used to map genetic variants to quantitative cell behavioral traits, which contributes to the growing number of iPSC-based cellular genetics studies investigating inter-individual heterogeneity in genomic, proteomic, and cellular traits during development, health, and disease (Bonder et al., 2021; Cuomo et al., 2020; Jerber et al., 2021; Mirauta et al., 2020; Vigilante et al., 2019).

Our platform can correlate altered protein function due to specific nsSNVs with altered cell behavior, as we have shown previously using iPSC lines in the pluripotent state (Vigilante et al., 2019). Nevertheless, it is likely that there are effects of nsSNVs that we fail to detect either through lack of sensitivity of the platform or because the nsSNVs manifest their effects in phenotypes that we have not measured. Without additional inference, the platform cannot distinguish between a gain-of-function or a loss-of-function variant, and may not resolve loss of functional activity of heterozygotes if one wild-type allele is sufficient for a normal phenotype or there is functional redundancy (Shawky, 2014).

Genetic or molecular markers that predict differentiation efficiency of iPSC lines would help advance their research and clinical applications. Indeed, previous studies have identified transcriptomic markers of differentiation capacity in iPSC lines (Cuomo et al., 2020; Jerber et al., 2021). A greater understanding of the mechanisms that influence differentiation propensity could help adapt culture conditions for more efficient differentiation protocols. For example, overexpression of WNT has been shown to improve endodermal differentiation in ESCs (Jiang et al., 2013).

Finally, our approach is a useful platform for functional genomics. Whole-genome sequencing is identifying genetic variants linked to psychiatric disorders and other forms of disease (Andrews et al., 2020; Sanders et al., 2017). To understand the mechanistic significance of those variants, simple surrogate *in vitro* assays based on cells harboring those variants are required. Our approach complements CRISPR interference-based screens (Kampmann, 2020) because it is not necessary to specifically target the genes/regulatory regions of interest. We therefore believe that the application of high-throughput quantitative cell-based assays and machine learning to genome-wide studies (Chandrasekaran et al., 2021) will find increasing applications in biomedical research.

## Experimental procedures

### iPSC line derivation and culture

iPSC lines were obtained from the HipSci cell bank at the Wellcome Trust Sanger Institute, Cambridge (www.hipsci.org; Kilpinen et al., 2017). The lines were derived from skin fibroblasts using Sendai virus vectors (CytoTune) expressing OCT4, SOX2, KLF4, and c-MYC. Quality control checks were performed including expression profiling to confirm pluripotency and genotyping arrays to detect copy number variation. All samples were obtained from consented research volunteers via the NIHR Cambridge BioResource (http://www.cambridgebioresource.org.uk) with approval from the UK National Health Service (NHS) Health Research Authority (REC 09/H0304/77, V2 04/01/2013; REC 09/H0304/77, V3 15/03/2013).

iPSCs were cultured on vitronectin (10 μg/mL, Stem Cell Technologies) in Essential 8 (E8) medium (Thermo Fisher) supplemented with 1% penicillin-streptomycin (Sigma). For routine maintenance, cultures underwent daily medium changes and were passaged every 4–5 days at approximately a 1:6 split ratio. Cell cultures were routinely tested for mycoplasma and all were negative for contamination. Details of the cell lines used are listed in Table S4.

### Genetic analysis

Gene Ontology analysis was performed using the web-services AmiGO 2 (http://amigo.geneontology.org/amigo/landing) and Gorilla (http://cbl-gorilla.cs.technion.ac.il/).

Rare SNVs were defined as those with a minor allele frequency (MAF) <0.005 in both the 1000 Genomes Project (1000 Genomes Project Consortium et al., 2012) and ExAC database and were present in fewer than five of the HipSci cell lines (Lek et al., 2016). SNVs were predicted to be deleterious to protein function based on the computational model Condel (González-Pérez and López-Bigas, 2011). Where structural information was available, the impact of SNVs on protein stability was predicted using the computational model DUET (Pires et al., 2014).

### Generation of micropatterned iPSC colonies

An adapted version of a previously described protocol was used to fabricate UV lithography micropatterned 96-well plates (Tewary et al., 2019). Briefly, 1000-μm diameter circular patterns were transferred onto custom sized (110 × 74 mm) coverslips by photo-oxidizing selected regions of the substrate using deep UV exposure (15 min) and glued to bottomless 96-well plates. Prior to cell seeding, wells were activated with N-(3-dimethylaminopropyl)-N′-ethlycarbodiimide hydrochloride and N-hydroxysuccinimide (20 min). After three washes with PBS, the wells were coated with 25 μg/mL fibronectin (Corning) overnight at 4°C. Immediately before seeding, the wells were washed four times with PBS to remove any passively adsorbed ECM protein.

iPSC colonies were incubated with TryplE (3 min, 37.5°C) and collected as a single-cell suspension in seeding medium (SM) consisting of 74% DMEM, 20% Knockout Serum Replacement (KOSR), 1% penicillin-streptomycin, 0.1 mM β-mercaptoethanol, 1% non-essential amino acids, 1% Glutamax, and 2% B27 minus retinoic acid, supplemented with 20 ng/mL basic fibroblast growth factor (bFGF) (all Thermo Fisher) and 10 μM ROCKi (Rho-associated protein kinase [ROCK] inhibitor [Sigma-Aldrich]). Cells were incubated with an anti-*ITGB1* antibody (P5D2; Byron et al., 2009) for 5 min prior to cell seeding where stated. Cells were seeded onto fibronectin-coated micropatterned 96-well plates at a density of 60,000 cells/well, unless otherwise stated. The cell line eojr_2 was seeded in two rows (20 wells) in the majority of plates to control for technical variation between experiments. Cells were incubated for 4 h (37.5°C), after which the medium was replaced with fresh SM supplemented with 20 ng/mL bFGF without ROCKi.

### Induction of germ layer differentiation

When cells had reached confluency (typically 15–20 h after seeding), germ layer differentiation was induced using N2B27 medium consisting of 93% DMEM, 1% penicillin-streptomycin, 0.1 mM β-mercaptoethanol, 1% non-essential amino acids, 1% Glutamax, 2% B27 minus retinoic acid, 1% N2 supplement, and supplemented with 50 ng/mL BMP4 (R&D), 100 ng/mL NODAL (R&D), and 10 ng/mL bFGF (Thermo Fisher). Cells were incubated for 48 h at 37.5°C prior to fixation.

### Immunofluorescence labeling

Cells were fixed using 4% paraformaldehyde (15 min, room temperature [RT]), permeabilized with 100% methanol (3 min, RT) then blocked using 5% donkey serum (30 min, RT). Primary antibodies diluted in 5% donkey serum were applied to wells overnight at 4°C. Following three washes with PBS, wells were incubated with secondary antibodies and DAPI (1:5,000) for 1 h at RT. Finally, wells were washed three times with PBS. All antibodies used are listed in Table S5.

### Imaging and analysis

Images were acquired using the Operetta CLS (PerkinElmer) microscope with a 20× 1.0 NA water objective and the Leica TCS SP8 confocal microscope with a 10× objective. An automated high-content image analysis pipeline was built in house using Harmony 4.5 software (PerkinElmer) to identify each micropatterned colony, select colonies quality controlled on area and roundness, determine the geometrical center of each colony, identify individual nuclei using the expression intensity in the DAPI channel, and measure the fluorescence intensity of each protein marker in each nucleus (see supplemental information). Single-cell data were exported from Harmony and analyzed using R 3.4.3. A script was written that divided each colony into 20 concentric rings spaced 25 μm apart, with nuclei assigned into the rings based upon their position relative to the colony center. Normalization of the fluorescence intensity data is described in the supplemental information. The data were used to (1) quantify the percentage of cells that expressed each germ layer marker per colony, and (2) compute the mean fluorescence intensity of each germ layer marker as a function of distance from the colony center.

Statistical analysis of mean protein expression (percentage of positive cells) across all cell lines was performed using the Kruskal-Wallis test with Dunn's multiple comparison post hoc test. Outlier cell lines based on mean protein expression (percentage positive) were identified by comparing the mean expression in one cell line with the mean of all cell lines pooled together using a two-tailed Student's t test, with p values <0.001 considered significant. Cell lines with outlier radial trends of protein expression were identified using the Kolmogorov-Smirnov test by comparing the radial protein expression in each cell line with the radial protein expression profile of the control iPSC line uoxz_4, with p values <0.01 considered significant. Spearman's rank correlation coefficients were calculated to analyze the relationship between expression of germ layer proteins. The Kruskal-Wallis test was performed in Prism. The Student's t test and Kolmogorov-Smirnov test were performed in R 3.4.3. Data are presented as the mean and standard deviation (SD).

## Author contributions

A.V., M.T., D.D., F.W., conceptualization; A.V., A.L., M.T., M.P., V.S., F.F., data curation; A.V., M.T., A.L., M.P., V.S., F.F., formal analysis; A.V., M.T., F.W., funding acquisition; A.V., investigation; A.V. and M.T., methodology; F.W., project administration; D.D. and F.W., supervision; A.V., M.T., A.L., M.P., and V.S., visualization; A.V. and F.W., writing – original draft; A.V., M.T., A.L., M.P., D.D., and F.W., writing – review & editing. All authors contributed to the article and approved the submitted version.

## Conflict of interests

F.M.W. is currently on secondment as Executive Chair, UK Medical Research Council.

D.D. is an employee of King's College London and an employee of bit.bio. D.D. declares no other affiliations with or involvement in any organization or entity with any financial or non-financial interest in the subject matter or materials discussed in this manuscript.

## Data Availability

The datasets and computer code produced in this study are available in the following databases:• Computer scripts: GitHub https://github.com/AliceVickers/pattern-profiler • Datasets: Figshare https://doi.org/10.6084/m9.figshare.14497725

## Supplemental information

Document S1. Supplemental experimental procedures, Figures S1–S5, and Tables S3–S5

Table S1. Protein expression profiles of SOX2, BRA and SOX17 were compared between technical and biological replicates in each individual cell line for the control iPSC lines uoxz_4, qanu_1, giuo_5, and eojr_2 using a Kolmogorov-Smirnov test, related to Figures 1D–1F and 2A–2C For the line uoxz_4, protein expression profiles of SOX2, BRA, and SOX17 were also calculated based on 85, 106, and 83 colonies, respectively. Randomly chosen subgroups of 4, 7, 10, or 20 colonies were selected and statistically compared with the overall colony profile using a Kolmogorov-Smirnov test.

Table S2. Details of iPSC lines with rare and deleterious SNVs in genes that regulate germ layer development and/or cell adhesion, related to Figure 2D

Document S2. Article plus supplemental information

## Footnotes

Supplemental information can be found online at https://doi.org/10.1016/j.stemcr.2021.09.017.
