# Supplementary material for: Plating human iPSC lines on micropatterned substrates reveals role for ITGB1 nsSNV in endoderm formation
Source: Stem Cell Reports. 2021 Oct 21;16(11):2628–41. doi: 10.1016/j.stemcr.2021.09.017 (PMC8581167; doi:10.1016/j.stemcr.2021.09.017)
Supplement: Document S1. Supplemental experimental procedures, Figures S1–S5, and Tables S3–S5 [file mmc1.pdf]

Stem Cell Reports, Volume *16*

Supplemental Information

Plating human iPSC lines on micropatterned substrates reveals role for *ITGB1* nsSNV in endoderm formation

Alice Vickers, Mukul Tewary, Anna Laddach, Martina Poletti, Vasiliki Salameti, Franca Fraternali, Davide Danovi, and Fiona M. Watt

A.

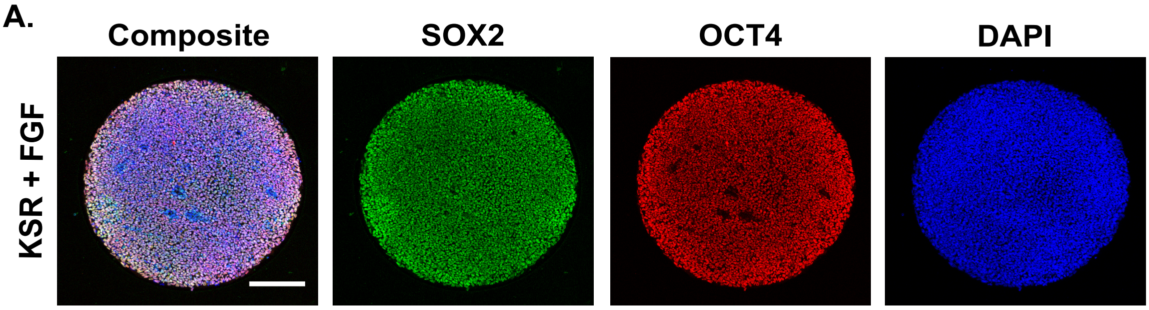

B.

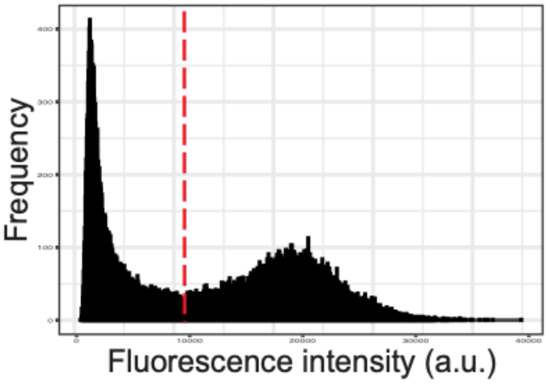

Figure S1. Related to Figure 1. *A)* iPSCs were seeded at a density of 60,000 cells/well on micropatterned substrates overnight. Cells were maintained in basal medium (KSR) with bFGF for 48h before fixation and stained with antibodies to detect SOX2 and OCT4, with DAPI counterstain. Representative confocal images are shown from *n*=3 experiments, each performed in triplicate (scale bar, 250μm). *B)* Intensity values were gated to remove those that represented background fluorescence by plotting histograms of the fluorescence intensity values for each well. The histograms separated fluorescence intensity values into two peaks, where the first peak corresponds to the background fluorescence. Values below the red dashed line were removed from subsequent analyses.

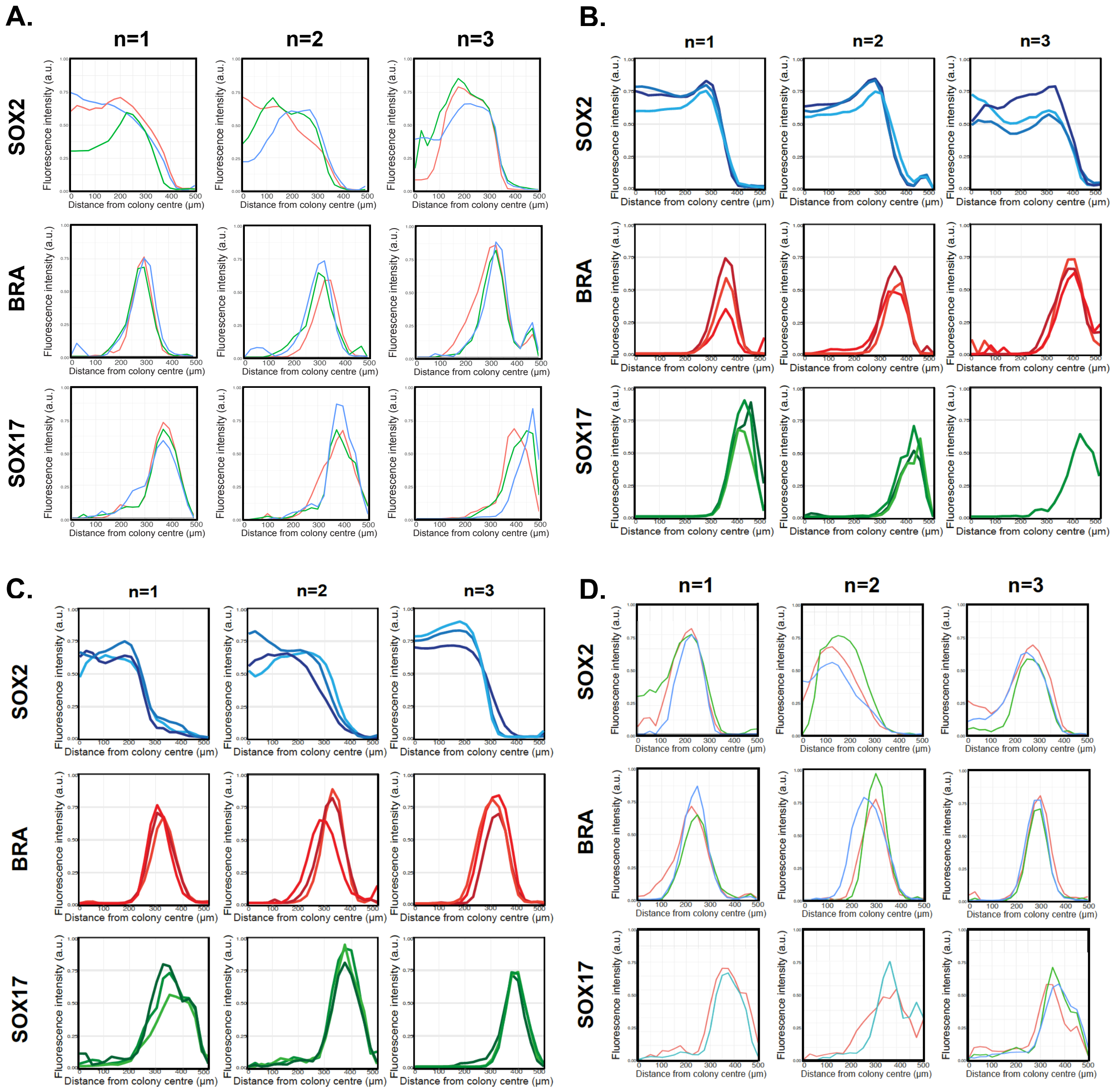

Figure S2. Related to Figures 1 & 2. Radial trend plots of SOX2, BRA and SOX17 expression for *A)* uoxz_4, *B)* qanu_1, *C)* giuo_5 and *D)* eojr_2 for each biological replicate (n). Each line on a plot represents data from one replicate (i.e. colonies averaged from one well).

<table>
<tr><th>Cell lines</th><th>Structure of protein impacted by nsSNV</th></tr>
<tr><td>yuze_1</td><td>ITGA6 (M50V)
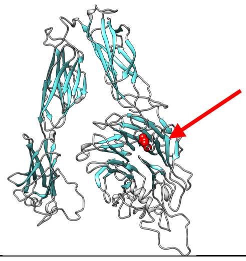
mCSCM predicted stability change (ΔΔG):
-1.004 kcal/mol
(destabilising)</td></tr>
<tr><td>pamv_1 & pamv_3</td><td>FHL2 (C272Y)
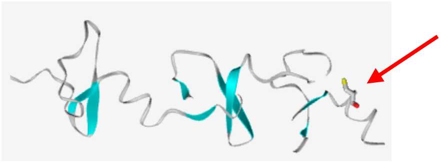
mCSCM predicted stability change (ΔΔG):
-1.148 kcal/mol
(destabilising)</td></tr>
<tr><td rowspan="2">ffdc_5 & ffdc_11</td><td>ITGB1 (S242F)
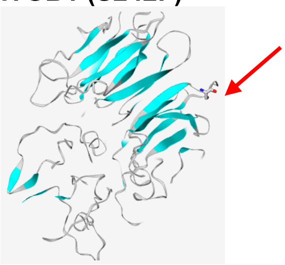
mCSCM predicted stability change (ΔΔG):
-1.428 kcal/mol
(destabilising)</td></tr>
<tr><td>TBXT (P280L)
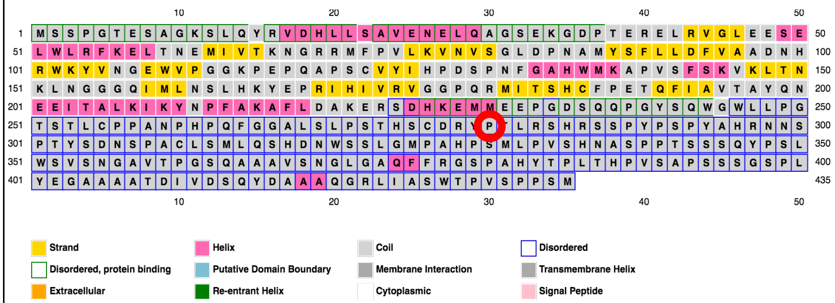
</td></tr>
<tr><td>lepk_1 & lepk_4</td><td>SMAD2 (G214S)
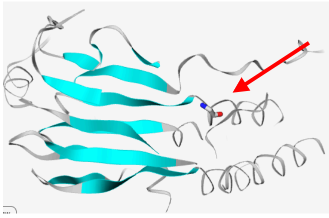
mCSCM predicted stability change (ΔΔG):
-1.568 kcal/mol
(destabilising)</td></tr>
<tr><td>bokz_5 & bokz_6</td><td>FGFR1 (P33L)
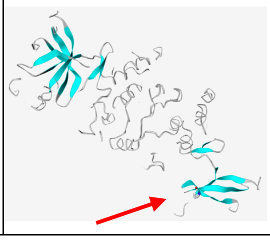
mCSCM predicted stability change (ΔΔG):
-0.325 kcal/mol
(destabilising)</td></tr>
</table>

Figure S3. Related to Figure 2D. Protein structures depicting the location of the identified nsSNVs in the test iPSC lines. The nsSNV in *TBXT* identified in ffdc_5 and ffdc_11 occurs in the disordered region of the protein, for which no 3D model was available.

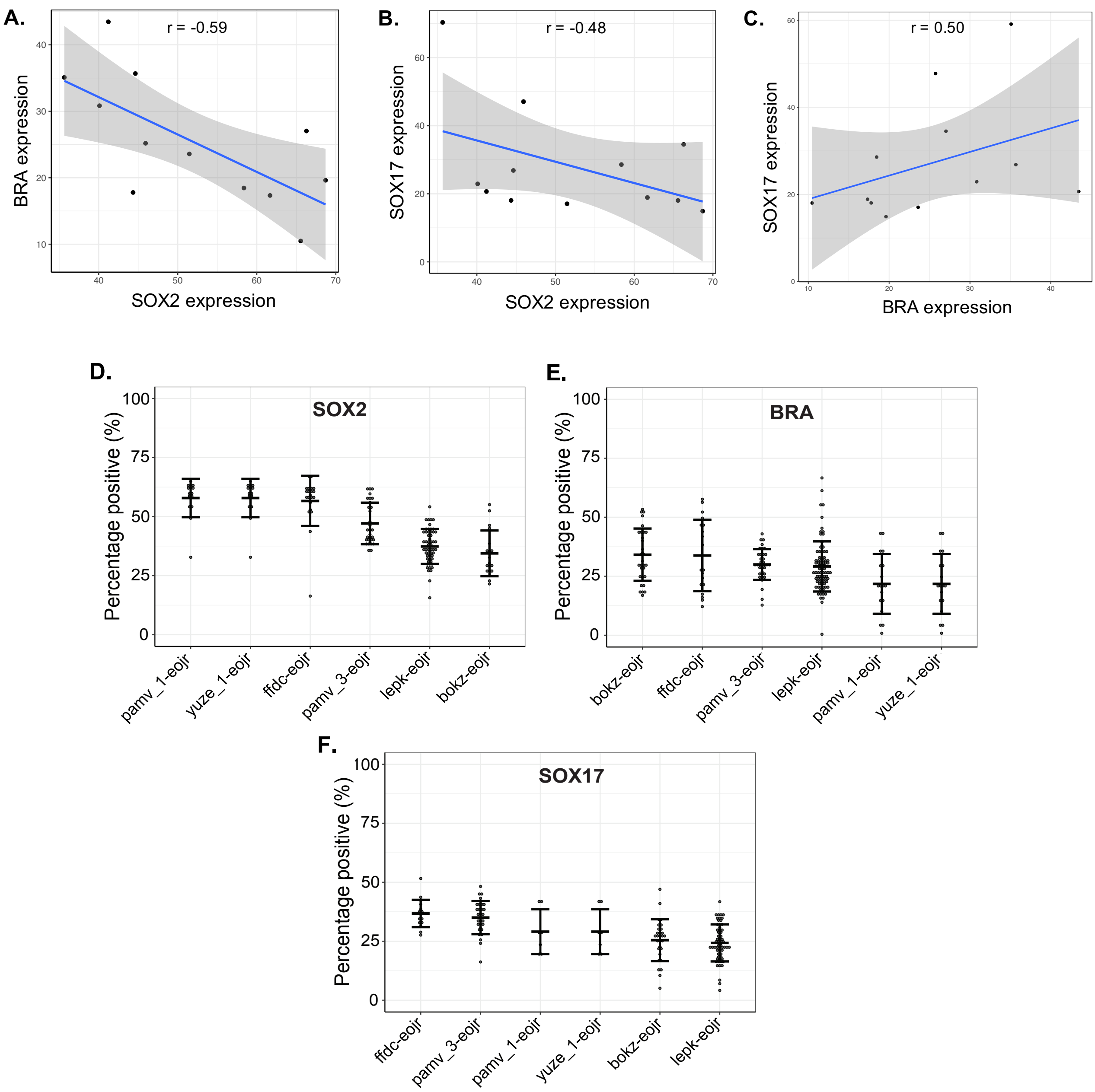

Figure S4. Related to Figure 3. Plots showing the correlation between *A)* SOX2 and BRA expression, *B)* SOX2 and SOX17 expression and *C)* BRA and SOX17 expression. r value represents Spearman's rank correlation coefficient. *D-F)* Quantification of protein expression for SOX2, BRA and SOX17 in the control iPSC line eojr_2, which was included in experiments involving biological replicates of the test iPSC lines. The relevant test iPSC line is indicated in the x-axis label. Each data point represents a micropatterned colony. Colonies were pooled from 2-3 independent experiments. Bars represent mean ±SD.

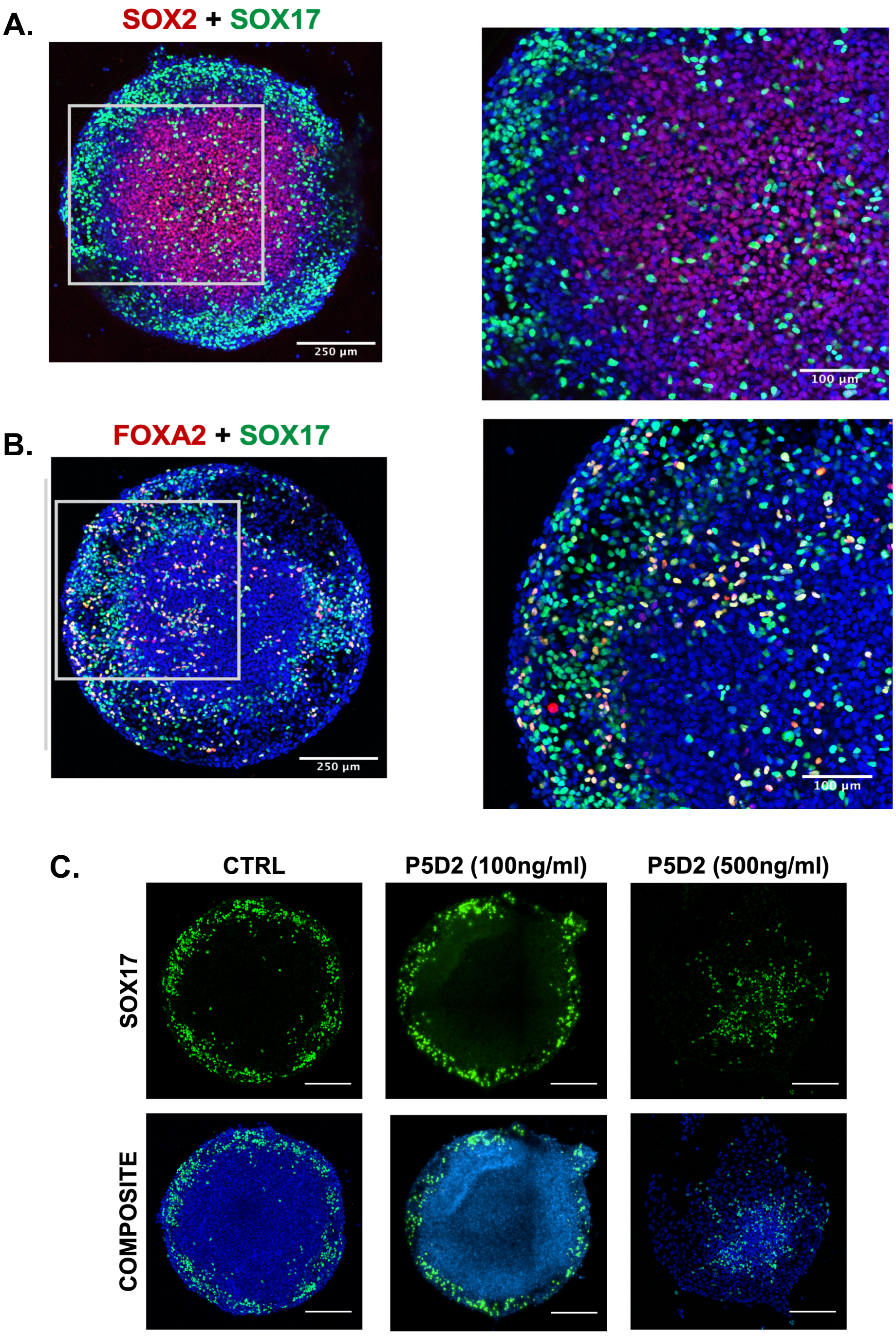

Figure S5. Related to Figure 4C & 5C. *A-B)* iPSCs of the line ffdc_11 were seeded at a density of 60,000 cells/well on micropatterned substrates overnight. Cells were treated with 50ng/ml BMP4 and 100ng/ml NODAL for 48h before fixation and stained with antibodies to detect *A)* SOX2 (red) and SOX17 (green), or *B)* FOXA2 (red) and SOX17 (green), plus DAPI (blue). Representative confocal images are shown from *n*=3 experiments, each performed in triplicate. Left hand images acquired using a 10x objective (scale bars, 250µm). Right hand images show boxed areas acquired using a 40x objective (scale bars, 100µm). *C)* Cell suspensions of the iPSC line uoxz_4 were incubated with 100ng/ml P5D2 or 500ng/ml P5D2 anti-ITGB1 antibody or remained untreated as a control (5 minutes, RT) and seeded at a density of 60,000 cells/well on micropatterned substrates overnight. Cells were treated with 50ng/ml BMP4, 100ng/ml NODAL and the same concentration of P5D2 antibody as before for 48h before fixation and stained with antibodies to detect SOX17 (green), plus DAPI (blue). Representative confocal images are shown from *n*=2 experiments, each performed in triplicate. Scale bars, 250µm.

Table S1. Related to Figures 1D-F & 2A-C. Protein expression profiles of SOX2, BRA and SOX17 were compared between technical and biological replicates in each individual cell line for the control iPSC lines uoxz_4, qanu_1, giuo_5 and eojr_2 using a Kolmogorov-Smirnov test. For the line uoxz_4, protein expression profiles of SOX2, BRA and SOX17 were also calculated based on 85, 106 and 83 colonies, respectively. Randomly chosen subgroups of 4, 7, 10 or 20 colonies were selected and statistically compared to the overall colony profile using a Kolmogorov-Smirnov test.

Table S2. Related to Figure 2D. Details of iPSC lines with rare and deleterious SNVs in genes that regulate germ layer development and/or cell adhesion.

Table S3. Related to Figures 2, 3 & 4. Number of colonies analysed per cell line for each germ layer protein**.**

Cell Line	SOX2	BRA	SOX17
pamv_1	89	99	80
pamv_3	88	77	90
yuze_1	36	34	25
ffdc_5	65	96	108
ffdc_11	60	55	49
bokz_5	24	20	21
bokz_6	70	76	63
lepk_1	52	100	98
lepk_4	61	62	61
uoxz_4	107	109	91
qanu_1	119	94	101
giuo_5	93	76	60

Table S4. Related to Experimental Procedures. Details of cell lines used in this study. Clonal iPSC lines from the same donor are denoted by the same 4 letter code with a unique number.

Name	Passage no. range	Culture conditions	Donor Characteristics			
			Age	Gender	Ethnicity	Disease Status
bokz_5	19-22	Feeder-free	55-59	Female	White British	Healthy
bokz_6	15-19					
debk_9	33-35	Feeder-free	Unknown	Female	Unknown	Healthy
eojr_2	20-24	Feeder-free	Unknown	Male	Unknown	Healthy
ffdc_5	25-29	Feeder-dependent	40-44	Male	Unknown	Healthy
ffdc_11	43-48	Feeder-free				
giuo_5	38-42	Feeder-free	Unknown	Male	Unknown	Healthy
lepk_1	22-26	Feeder-free	60-64	Female	White British	Healthy
lepk_4	16-19					
oikd_2	18-21	Feeder-free	40-44	Female	White British	Healthy
oikd_5	15-18					
pamv_1	30-34	Feeder-free	65-69	Male	White British	Healthy
pamv_3	15-18					
qanu_1	26-29	Feeder-free	Unknown	Female	Unknown	Healthy
toss_1	25-28	Feeder-free	65-69	Male	White British	Healthy
toss_3	25-28					
uoxz_4	18-22	Feeder-free	Unknown	Female	Unknown	Healthy
vils_1	15-17	Feeder-free	35-39	Female	White British	Healthy
yuze_1	37-40	Feeder-free	Unknown	Male	Unknown	Healthy

Table S5. Related to Experimental Procedures. Antibodies used in this study.

Antibody	Catalogue Number	Dilution
Goat anti-SOX2	R&D Systems AF2018-SP	1:200
Goat anti-BRACHYURY	R&D Systems AF2085	1:200
Goat anti-SOX17	R&D Systems AF1924	1:200
Rabbit anti-OCT4	Abcam ab19857	1:500
Mouse anti-Integrin beta 1 (P5D2)	Abcam ab 24693	400ng/ml
Donkey anti-goat Alexa Fluor 488	ThermoFisher A-11055	1:500
Donkey anti-rabbit Alexa Fluor 555	ThermoFisher A-31572	1:500

Supplemental Experimental Procedures

Harmony® Image Analysis Pipeline

Input Image
Flatfield Correction: None
Brightfield Correction: Yes
Stack Processing: Maximum Projection
Create Global Image: Yes
Min. Global Binning: Dynamic

Find Image Region
Channel: DAPI (global)
ROI: Imaged Area (global); Imaged Area
Method: Absolute Threshold

- Lowest intensity > 500
- Highest intensity < INF

Split into Objects: Yes
Output Population: Colonies
Output Region: Image Region

Find Surrounding Region
Channel: DAPI (global)
Population: colonies
Region: Image Region
Method: B
Output Region: Regions

Modify Population
Population: Colonies
Region: Regions
Method: Cluster by Distance
Distance: 5px
Area: >10000px^2
Output Population: Modified colonies
Output Region: Region

Select Population
Population: Modified colonies
Method: Common Filters
Remove Border Objects: Yes
Output Population: Modified colonies Selected

Calculate Morphology Properties (2)
Population: Modified colonies Selected
Region: Region
Method: Standard
Area: Yes µm^2
Roundness: Yes
Width: No
Length: No
Ratio Width to Length: No
Property Prefix: Region

Selection Population (2)
Population: Modified colonies Selected
Method: Filter by Property
Region area [µm^2] > 720000
Region area [µm^2] < 960000
Region roundness > 0.5

Output Population: Gastruloids

Select Region
Population: Gastruloids
Region: Region
Method: Standard
Border: Yes
Filled Region: No
Geometrical Center: Yes
Region Prefix: Gastruloid centre

Find Nuclei
Channel: DAPI (global)
ROI: Gastruloids; Region
Method: M
Output Population: Nuclei_within_gastruloids

Calculate Intensity Properties
Channel: Alexa 488 (global)
Population: Nuclei_within_gastruloids
Region: Nucleus
Method: Standard
Property Prefix: Intensity Nucleus Alexa 488 (global)

Calculate Intensity Properties (2)
Channel: DAPI (global)
Population: Nuclei_within_gastruloids
Region: Nucleus
Method: Standard
Property Prefix: Intensity Nucleus DAPI (global)

Normalisation of fluorescent intensity data
Within each nucleus, the fluorescence intensity of each protein marker was normalised to DAPI intensity. The fluorescence intensity values were gated to remove those that represented background fluorescence by plotting histograms of the fluorescence intensity values for each well. The histograms separated fluorescence intensity values into two peaks, where the first peak of low fluorescence intensity values was due to background fluorescence and thus these values were removed from subsequent analyses (Figure S1B). These values were then normalised to the maximum expression value within the well.
